# The role of angiogenesis in implant dentistry part I: Review of titanium alloys, surface characteristics and treatments

**DOI:** 10.4317/medoral.21199

**Published:** 2016-03-31

**Authors:** Mohammad-Ali Saghiri, Armen Asatourian, Franklin Garcia-Godoy, Nader Sheibani

**Affiliations:** 1 Bsc, Msc, PhD. Departments of Ophthalmology & Visual Sciences, Biomedical Engineering, and McPherson Eye Research Institute, University of Wisconsin School of Medicine and Public Health, Madison, WI ,USA; 2 DDS. Sector of Angiogenesis and Regenerative Surgery, Dr.H Afsar Lajevardi Cluster, Shiraz, Iran; 3 DDS, MS, PhD, PhD. Bioscience Research Center, College of Dentistry, University of Tennessee Health Science Center, TN, USA

## Abstract

**Background:**

Angiogenesis plays an important role in osseointegration process by contributing to inflammatory and regenerative phases of surrounding alveolar bone. The present review evaluated the effect of titanium alloys and their surface characteristics including: surface topography (macro, micro, and nano), surface wettability/energy, surface hydrophilicity or hydrophobicity, surface charge, and surface treatments of dental implants on angiogenesis events, which occur during osseointegration period.

**Material and Methods:**

An electronic search was performed in PubMed, MEDLINE, and EMBASE databases via OVID using the keywords mentioned in the PubMed and MeSH headings regarding the role of angiogenesis in implant dentistry from January 2000-April 2014.

**Results:**

Of the 2,691 articles identified in our initial search results, only 30 met the inclusion criteria set for this review. The hydrophilicity and topography of dental implants are the most important and effective surface characteristics in angiogenesis and osteogenesis processes. The surface treatments or modifications of dental implants are mainly directed through the enhancement of biological activity and functionalization in order to promote osteogenesis and angiogenesis, and accelerate the osseointegration procedure.

**Conclusions:**

Angiogenesis is of great importance in implant dentistry in a manner that most of the surface characteristics and treatments of dental implants are directed toward creating a more pro-angiogenic surface on dental implants. A number of studies discussed the effect of titanium alloys, dental implant surface characteristic and treatments on agiogenesis process. However, clinical trials and *in-vivo* studies delineating the mechanisms of dental implants, and their surface characteristics or treatments, action in angiogenesis processes are lagging.

**Key words:**Angiogenesis, dental implant, osseointergration.

## Introduction

In the field of implant dentistry, titanium and its alloys are one of the most commonly used groups of materials due to their unique biocompatibility, mechanical characteristics, and chemical stability. These distinct values of titanium are because of the formation of an oxide layer on the surface, which has a superior role in establishing a direct contact between dental implant and the surrounding alveolar bone, known as osseointegration (1-3). In addition to TiO2 layer, other elements are effective on osseointegration including the titanium implant surface composition and topography (4). The dental implants, which have micron-scale surface roughness could better induce the osteoblast culture and differentiation compared to implants with machined surfaces (5,6). Even in normal bone formation, osteoclasts make the surface roughness, which plays a critical role for osteoblasts activity such as differentiation, mineralization, and production of growth factors like transforming growth factor beta-1 (TGF-β1) and bone morphogenetic proteins (BMPs) (7-9).

Angiogenesis is the formation of new blood vessels from pre-existing capillaries, with major contribution to inflammatory and regenerative events in the body’s tissues including bones. The critical role of angiogenesis in regenerative dental procedures, which deal with dentin-pulp complex and dental pulp regeneration has been recently discussed (10,11). The formation of new bones, bone regeneration, and also osseointergration after dental implant installation require a blood supply to provide nutrition, oxygen, and osteoprogenitor cells through the newly formed blood vessels (11-13). Authors have previously indicated that there is an interaction between angiogenesis and osteogenesis, in a way that the regulation of angiogenesis plays a crucial role in bone remodeling through the wound healing process (14).

According to the close relationship between angiogenesis, osteogenesis, and osseointergration, in this review we provide an overview of the role of titanium alloys (titanium-aluminum-vanadium) and dental implant surface characteristics including surface topography (macro, micro, and nano), surface wettability/energy, surface hydrophilicity or hydrophobicity, surface charge, and treatments on angiogenesis processes occurring in surrounding alveolar bone after dental implant installation.

## Material and Methods

1. The Review Purpose:

The present review was conducted to assess the effects of titanium alloys and dental implant surface characteristics and treatments on angiogenesis processes that occur during peripheral alveolar bone regeneration. In order to assess these effects, the following main aspects were selected according to the aims of this review. These aspects included: 1) the effects of titanium and its alloys used in implant dentistry on angiogenesis processes; 2) the pro-or anti-angiogenic properties of these elements and their mechanisms of action; 3) the effect of dental implant surface characteristics on angiogenesis processes; 4) identify the surface characteristic or characteristics that have the highest pro-angiogenic effect; 5) the effect of dental implant surface treatments on angiogenesis processes; 6) identify the surface treatment or treatments that have the greatest pro-angiogenic impact; and 7) the current state and trend of dental implant surface treatments from the angiogenesis point of view.

2. Search Strategy for the Identification of Pertinent Studies:

The searching methodology included electronic searches performed in the PubMed, MEDLINE, and EMBASE databases via OVID using keywords mentioned in the PubMed and MeSH headings including the impacts of titanium alloy used in implant dentistry and the dental implant surface characteristics and treatments on angiogenesis events beginning in alveolar bone after insertion of root forming dental implants. In the electronic search of scientific papers in the PubMed, MEDLINE, and EMBASE databases, the following keywords were used in combination with angiogenesis: “Titanium alloys and angiogenesis”, “dental implant and angiogenesis” dental implant surface topography”, “dental implant surface wettability”, “dental implant surface energy”, “ dental implant surface hydrophilicity”, “dental implant surface hydrophobicity”, “dental implant surface charge”, and “machined surface dental implant”, “sand-blast large grit acid etched dental implant surface”, “acid-etched dental implant surface”, “dual acid-etched dental implant surface”, “anodized/oxidized dental implant surface”, “plasma-sprayed dental implant surface”, “bisphosphonate coated dental implant”, “simvastatin coated dental implant”, “antibiotic coated dental implant”, “functionalized dental implant”, “cytokine coated dental implant”, and “growth factor coated dental implant”.

3. Inclusion and Exclusion Criteria.

The inclusion criteria were: 1) studies published in English; 2) studies accepted and published between January 2000-April 2014; 3) the scientific *in-vitro* or *in-vivo* articles, reviews, systematic reviews, case reports that had evaluated the effect of titanium and it alloys, used in implant dentistry, on angiogenesis processes; and 4) studies that had evaluated and compared the effect of different dental implant surface characteristics and treatment on angiogenesis processes. The exclusion criteria were: 1) the studies that were not published between January 2000 and April 2014; 2) the studies that only focused on the effect of titanium surface characteristics or treatments on osteogenesis or osseonintegration.

## Results

Of the 2,691 articles found in initial search results, only 30 met the inclusion criteria set for this review. These 30 studies were directly related to the effect of dental implant titanium alloys, surface characteristics, and treatments on the angiogenesis processes, which are presented in  Table 1 and  Table 1Continue . The relevant full text articles and the reference lists of the related articles were evaluated to supplement the search as well. The assessment of the eligibility and finding related data were performed by two reviewers independently. A third reviewer was selected for further discussion and final agreement on any conflict met in the mentioned processes.

**Table 1:**
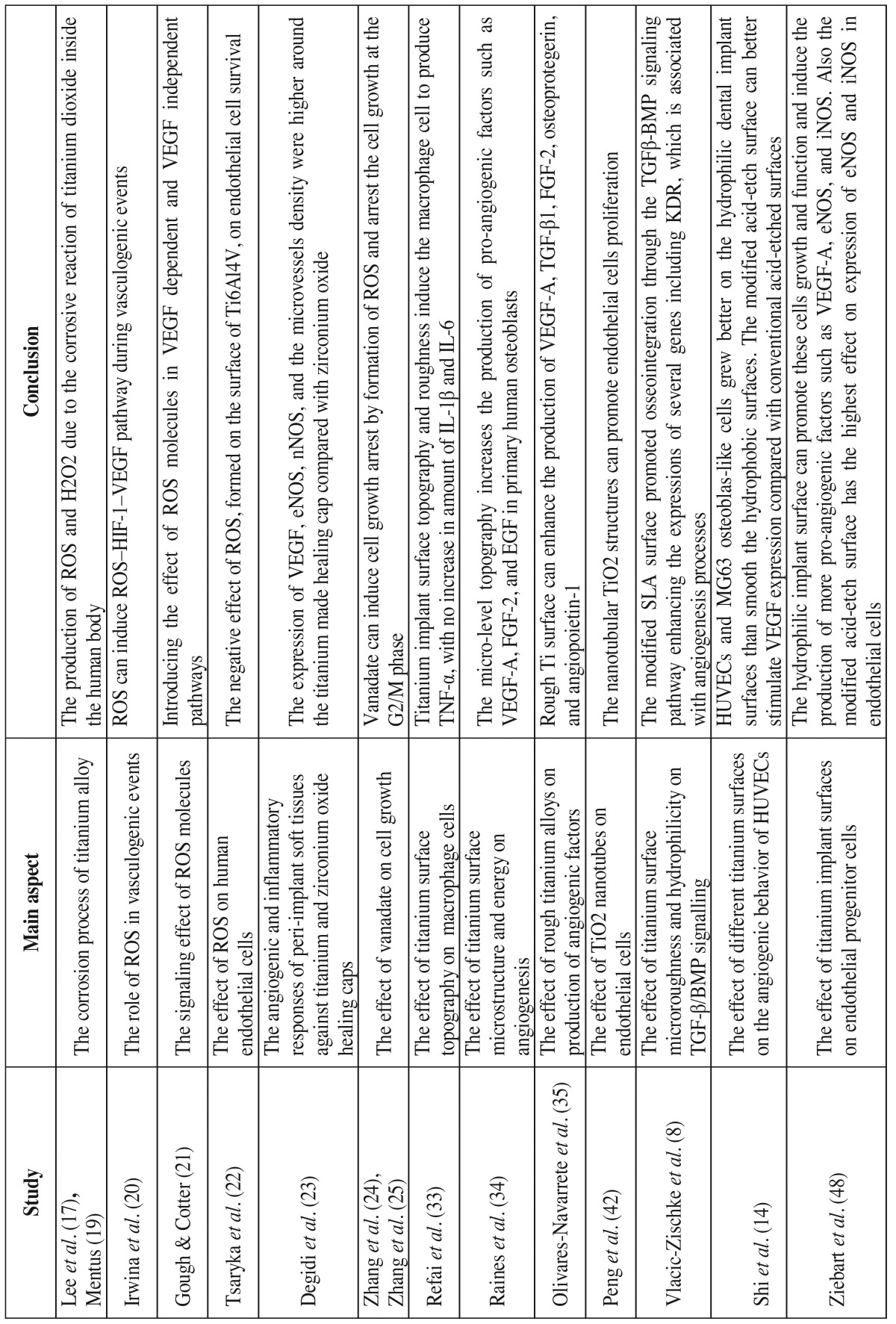
The list of included studies that are directly related to the effect of dental implant titanium alloys, surface characteristics, and treatments on angiogenesis process.

**Table 1Continue:**
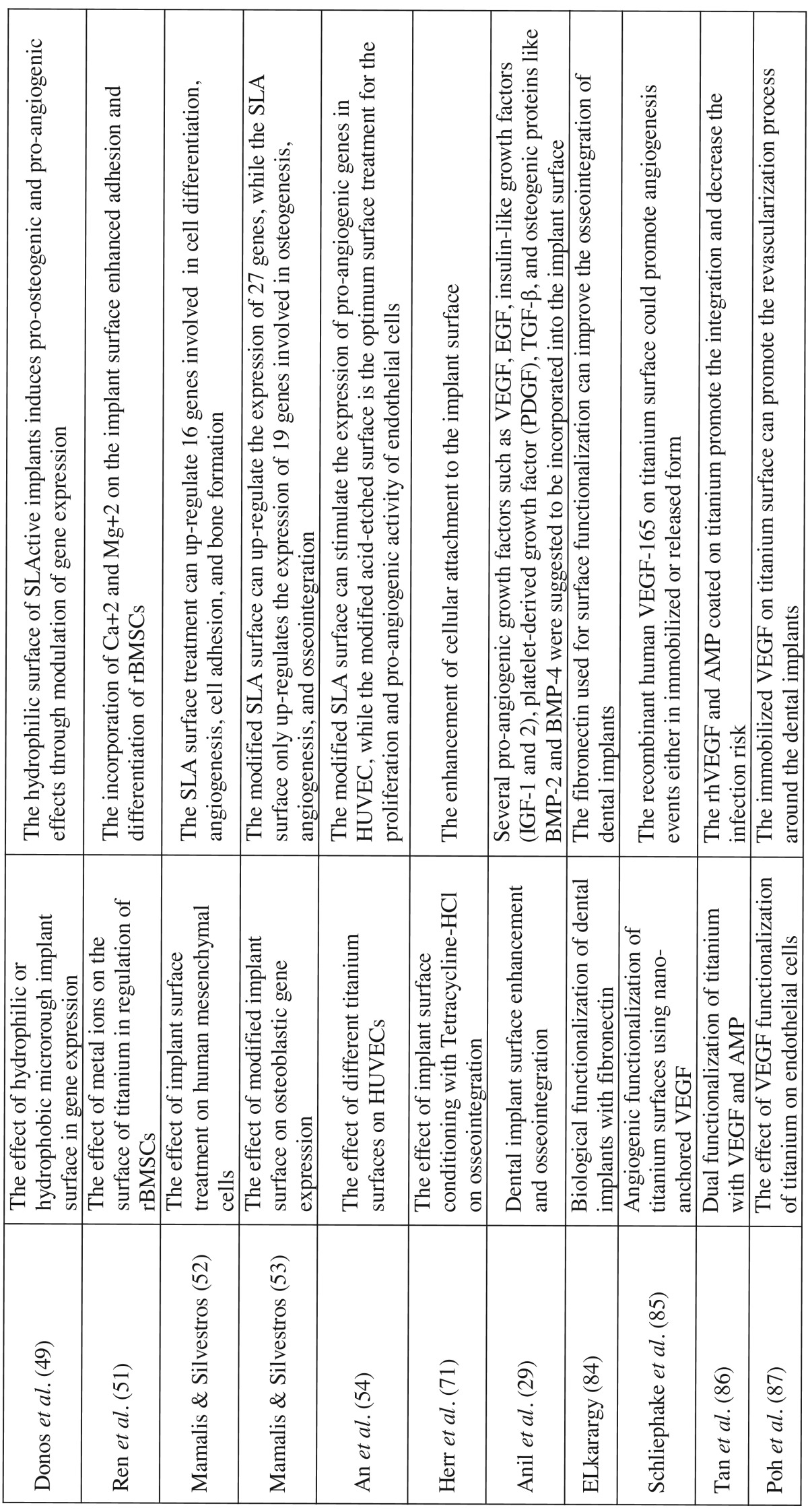
The list of included studies that are directly related to the effect of dental implant titanium alloys, surface characteristics, and treatments on angiogenesis process.

## Discussion

1 Effects of titanium alloys on angiogenesis:

Titanium and its alloys are load-bearing metals, which are used in manufacturing bio-implants. The titanium made implants, due to their TiO2 layer have a unique ability to establish a reliable attachment to surrounding bone structure (3). The titanium alloy used for manufacturing dental implants is the alloy of Ti6Al4V, which is covered by a TiO2 passivating layer. This layer is well attached to the surface and acts as a protective layer on the surface of titanium alloys. This layer may be the main reason for the high biocompatibility of this alloy (1), since the biological systems sense the surface of biomaterials (15,16). However, several investigators indicated that the surfaces of titanium alloys are reactive in different environments including inside the human body (17,18). Hallab *et al.* reported that the serum titanium concentration increases in patients after total knee replacement surgery during the first month (18). Mentus introduced two corrosion processes on the titanium alloy surfaces including anodic and cathodic corrosion (19). The anodic corrosion occurred in areas where there were defects in the passivating oxide layer (TiO2). In the cathodic process, the reduction of oxygen occurs resulting in the formation of intermediate products such as reactive oxygen species (ROS) and hydrogen peroxide (H2O2) (19). In addition to the ROS formed by the corrosion process, the inflammatory cells such as macrophages, monocytes, and granulocytes can also produce ROS (17).

ROS are important molecules with remarkable role in angiogenesis events. Irwin *et al.* indicated that ROS are involved in the ROS–HIF-1–VEGF pathway during hypoxia induced angiogenesis (20). Gough, Cotter introduced different mechanisms that ROS molecules can present proangiogenic activities (21). The other possible pathway of ROS activity is known as the VEGF-independent pathway. In this pathway, the main role is played through modulation of the activity of transcriptions factors such as AP-1 and NF-κB ( Table 1 and  Table 1Continue ,  Table 2).

**Table 2:**
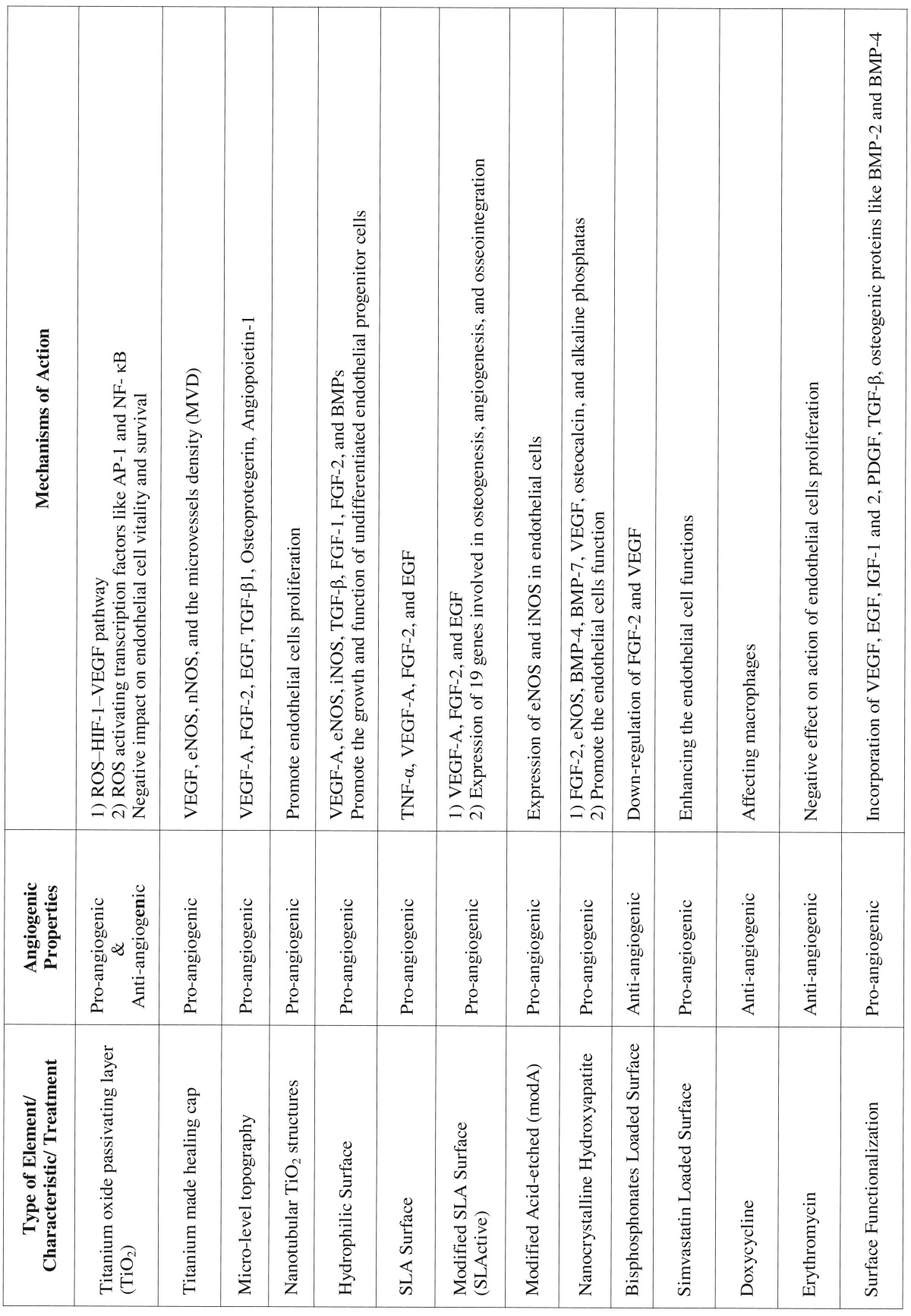
The angiogenic properties of dental implant titanium alloy elements, characteristics, and treatments.

Despite the prominent roles of ROS in angiogenesis events, Tsaryk *et al.* indicated that ROS formation on the surface of titanium alloy (Ti6Al4V) can negatively impact endothelial cell survival (22). These authors indicated that the increased production of ROS and oxidative stress can adversely affect the vitality of endothelial cells and attenuate the angiogenesis process (22). Degidi *et al.* compared the angiogenic and inflammatory responses of peri-implant soft tissues to the titanium and zirconium oxide healing caps (23). These authors indicated that the expression of VEGF, endothelial nitric oxide synthase (eNOS or NOS3), neuronal nitric oxide synthase (nNOS or NOS1), and the microvessels density were higher around the titanium made healing cap compared with zirconium oxide (23) ( Table 1 and  Table 1Continue ,  Table 2). Other compositions of titanium alloy such as vanadium was able to produce ROS (24). Zhang *et al.* reported that vanadate can induce cell growth arrest by formation of ROS, which can affect mitogen-activated protein kinases family members and arrest the cell growth at the G2/M phase (25) ( Table 2).

2-1. Effect of surface characteristics on angiogenesis:

Osseointergration is defined as a direct structural and functional contact between the bone and the load-bearing implant. This connection is crucial for stability, loading, and long-term survival of dental implants (3) and biomaterials (26). Titanium and its alloys are not capable of establishing a reliable bone-to-implant contact, and they need surface modifications to enhance osseoingtegration (27). Sul *et al.* indicated that osseointegration is closely related to the chemical and physical characteristics of titanium made implants (27). These characteristics mainly include the surface topography and roughness (28), wettability, surface energy, charge, surface chemistry, chemical potential, metal or non-metal surface composites, impurities, and thickness of TiO2 layer (29,30). Among these characteristics, the surface topography and roughness, wettability/energy, hydrophilicity or hydrophobicity, and surface charge had the greatest impact on osseointegration (29). Thus, in the following section we review the impacts of most effective and important characteristics of dental implant surface on angiogenesis events involved in osteogenesis process and osseointegration.

2-2. Surface macro- and micro-scale topographies and roughness:

Dental implant surface topography is described as the macroscopic and microscopic textures (31). Shibli *et al.* reported that different surface textures and topographies can induce proliferation, transformation, and function of osteoblasts (32). Dental implant surface roughness is divided into three types of macro-, micro-, and nano-roughness according to the dimensions of the surface texture (29). Macro-roughness is referred to the surface features with dimensions between microns to millimeters, and in micro-roughness between 1-10 microns (29). Refai *et al.* (33) demonstrated that the titanium implant surface topography and roughness created by sandblasted large grit acid etched and acid etching (AE) treatments stimulated the macrophages to secrete proinflammatory cytokine including tumor necrosis factor (TNF)-α, which plays a great role in angiogenesis events immediately after implantation. Meanwhile, they reported that the macrophages’ secretion of interleukin IL-1β and IL-6 were not increased, and the production of chemokines like the monocyte chemoattractant protein (MCP)-1 and macrophage inflammatory protein (MIP)-1α were down-regulated after culture on sand-blasted large grit acid etched and AE surfaces (33). However, these investigators reported that macrophages stimulated by lipopolysaccharide (LPS) could produce higher levels of these cytokines (TNF-α, IL-1β, IL-6) and chemokines (MCP-1, MIP-1α) (33) ( Table 1 and  Table 1Continue ,  Table 2). Raines *et al.* showed that the micro-level topography increases the production of pro-angiogenic factors such as VEGF-A, fibroblast growth factor (FGF)-2, and epidermal growth factor (EGF) in primary human osteoblasts (HOB) through α2β1 signaling pathway (34). Olivares-Navarrete *et al.* reported similar results and indicated that rough Ti surface can enhance the secretion of VEGF-A, TGF-β1, FGF-2, osteoprotegerin, and angiopoietin-1 by osteoblast-like MG63 cells (35) ( Table 1 and  Table 1Continue).

Vandamme *et al.* pointed out that the mechanical loading on dental implants has greater effect on osseointegration process than the microtopography of implant surface (36). These authors indicated that in case of load-free implants, the surface micro-roughness enhanced the osteogenic activity better than turned or machined surfaces. However, in load-bearing or loaded implants this surface characteristic did not show significant enhancement in oseointegration compared with machined surface implants (36).

2-3. Surface nano-scale topographies and roughness:

The nano-scale topographies are the features on dental implant surfaces with dimensions ranging between 1-100 nanometers (29,37). The rationale for making dental implants with nano-roughness on the surface was to increase the surface texture and topography. This issue can result in increased surface energy and wettability, which accelerates the adhesion of matrix proteins, growth factors, and osteoblasts (29,38). Several authors investigated the effect of nano-roughness or nanostructure of dental implant surface during osseointegration. Webster *et al.* (39) and Dalby *et al.* (40) reported that the nanotopographies of dental implants modulate the cell adhesion and osteoblast cells differentiation. The effects of nano-scale topographies on different cell types such as osteoblasts, fibroblasts, epithelial cells, and myocytes have been discussed (41). There are few studies which directly investigated the effects of nanotopographies of titanium surface on the endothelial cells function and angiogenesis events (42,43). Peng *et al.* indicated that the nanotubular TiO2 structures can promote endothelial cells proliferation and increase the secretion of prostaglandin I-2 (PGI-2) (42) ( Table 1 and  Table 1Continue ). Jo *et al.* indicated that TiO2 nano particles can be used for treatment of renal diseases due to their anti-angiogenic properties (43) ( Table 2).

2-4. Surface wettability and energy:

The wettability of dental implant surface is one of the most important surface characteristics for osseointegration (29). The higher energy or wettability of titanium implant surface can change its interactions with surrounding cells. These interactions are influenced by the better adsorption of protein molecules to the implant surface with high wettability (41).

Rupp *et al.* showed that wettability of implant surface is of great importance and can be modified by other implant surface characteristics, especially surface topography and chemistry (44). This issue was also emphasized by Elias *et al.*where they showed that implant surface wettability was closely related to the surface roughness and morphology (45). In 1985, Van Wachem *et al.* investigated the effect of wettability of different polymers on function of human endothelial cells, and concluded that polymers with greater wettability, due to better adsorption of serum and/or cellular protein molecules, can promote the growth of human endothelial cells (46).

2-5. Surface hydrophilicity and hydrophobicity:

The hydrophilicity or hydrophobicity of dental implant surface is crucial determinant for interactions with blood at inserted sites. After surgical installation of dental implants in alveolar bone, the hydrophilic surface of implant can promote the coagulation of blood and adsorption of matrix proteins such as fibronectin. The process of oseeointergration begins with the migration and function of macrophages in the subjected area. These immune cells, after removal of necrotic debris resulted from surgical phase, can produce several growth factors including TGF-β, FGF-1, FGF-2, and BMPs that are pro-osteogenesis and pro-angiogenesis (29) ( Table 1 and  Table 1Continue).

Numerous attempts have been made to modify the surface of dental implants to make them more hydrophilic. Vlacic-Zischke *et al.* indicated that the hydrophilic sand-blasted acid-etched surface showed better osseointegration than unmodified sand-blasted large grit acid etched surface (8). These investigators reported that the modified sand-blasted large grit acid etched surface promoted osseointegration through the TGFβ-BMP signaling pathway enhancing the expressions of several genes including KDR (VEGF-R2), which is associated with angiogenesis processes (8). Shi *et al.* indicated that the co-culture of human umbilical vein endothelial cells (HUVEC) and MG63 osteoblas-like cells grew better on the hydrophilic dental implant surfaces than the smooth hydrophobic surfaces (47). Ziebart *et al.* also addressed this issue and showed that hydrophilic implant surface can promote growth and function of undifferentiated endothelial progenitor cells to produce more pro-angiogenic factors including VEGF-A, eNOS, and iNOS (48). Donos *et al.* showed that hydrophilic sand-blasted acid-etched surface of dental implants compared with sand-blasted large grit acid etched surface, imposed more pro-osteogenic and pro-angiogenic effects through modulation of gene expression (49). Furthermore, Schwarz *et al.* reported that the hydrophilicity of dental implant surface is a more important factor than the surface microtopography in soft and hard tissue integration (50).

2-6. Surface charge:

The dental implant surface charge is modified by chemical treatment methods, which usually use sodium or potassium hydroxide followed by heat treatment. The result of this treatment is the formation of titanium hydroxide (Ti-OH) on the surface of implant, which exchanges the sodium ions with the calcium ions present in the surrounding tissue (29). Ren *et al.* demonstrated that the incorporation of Ca+2 and Mg+2 on the implant surface enhanced the adsorption of proteins, which was followed by adhesion and differentiation of bone-marrow derived stem cells (51) ( Table 2).

3. Effect of surface treatments on angiogenesis:

The surface treatments or modifications of dental implants are performed to induce and alter their characteristics. The majority of these treatments are performed to generate different topographies or roughnesses on the surface. However, other characteristics such as wettability, surface energy, and surface charge are also impacted by the surface modifications (2,16,29). Basically, there are three types of methods for surface treatments, including physical, mechanical, and chemical, which enhance the ostegenesis and osseointegration. The common mechanical methods are machining, polishing, blasting, and grinding, which produce either smooth or rough surfaces in order to promote the adhesion, proliferation, and differentiation of surrounding cells (29).

The other common method is the chemical treatment, which removes the surface contaminants and increases the biomechanical properties that result in enhanced osseointegration. Surface treatments with hydrogen peroxide (H2O2), acids, alkali, anodization, chemical vapor deposition, and sol-gel are different types of chemical modification. The last method is a physical method including plasma spraying, ion deposition, and sputtering methods (29). In the following sections, the effects of most common surface treatments on angiogenesis events are overviewed.

3-1. Machining or turning.

The first treatment method of dental implant surface was the machining or turning, which creates a polished surface. This is one of the physical treatment methods, which was used for first generation of implants (29). Thus far, the effect of this treatment on angiogenesis events has not been evaluated.

3-2. Sand-blasting large grit acid-etched.

Another common physical method used for surface treatment of dental implants is the grit blasting, which is done by titanium or aluminum particles ranging in size between 25-75 µm. The sandblasting and acid-etching treatment method includes the grit blasting with 250-500 µm size particles, and the acid-etching is done with hydrochloric and sulfuric acid (29). The sand-blasted large grit acid etched surface treatment was demonstrated to induce macrophages to produce TNF-α, a pro-angiogenic factor 33. Similarly the expression of other pro-angiogenic factors such as VEGF-A, FGF-2, and EGF are increased on sand-blasted large grit acid etched or modified sand-blasted large grit acid etched treated dental implants (34) ( Table 1 and  Table 1 Continue).

Several authors have compared the pro-angiogenic and pro-osteogenic activity of conventional unmodified sand-blasted acid-etched and the modified sand-blasted large grit acid etched surface that is hydrophilic. These investigators presented similar results that the modified sand-blasted large grit acid etched surface known as hydrophilic sand-blasted acid-etched can better stimulate the adsorption of proteins, alveolar bone stem cells differentiation, and result in a better osseointegration and angiogenesis in surrounding bone (8,48,49). Mamalis, Silvestros showed that the sand-blasted large grit acid etched surface treatment can up-regulate 16 genes, which are effective in cell differentiation, angiogenesis, cell adhesion, and bone formation (52). These authors in another study showed that the modified sand-blasted large grit acid etched surface can up-regulate the expression of 27 genes, while the sand-blasted large grit acid etched surface only up-regulates the expression of 19 genes involved in osteogenesis, angiogenesis, and osseointegration (53). Shwarz *et al.* demonstrated that the modified sand-blasted large grit acid etched surface, compared to machined sand-blasted large grit acid etched, could better promot the soft and hard tissue integration (50). An *et al.* showed that modified sand-blasted large grit acid etched surface can stimulate the expression of pro-angiogenic genes in HUVEC (54) ( Table 2).

3-3. Acid-etching (AE) and dual acid-etching:

The acid-etching is the most common chemical method used for surface treatment. The acid-etching is usually performed by either hydrochloric acid (HCl) and sulfuric acid (H2SO4), or nitric acid (HNO3) and hydrofluoric acid (HF). The duel acid etching is performed by heated HCl and H2SO4 (29). Mamalis, Silvestros indicated that the dual-acid etched (Osseotite) surface of dental implants can up-regulate the expression of 15 genes involved in osteogenesis and angiogenesis (52). An *et al.* reported that among acid-etched (A), sand-blasted large grit acid etched, modified acid-etched (modA), and modified sand-blasted large grit acid etched (modSLA) the modA is the optimum surface treatment for the proliferation and pro-angiogenic activity of endothelial cells (54). Similar results were reported by Ziebart *et al.*, which indicated that the modA has the highest effect on expression of eNOS and iNOS in endothelial cells, while the conventional acid-etched surface showed the lowest stimulation (48). Shi *et al.* showed that modA surface can better stimulate VEGF expression compared with conventional acid-etched surfaces (14).

3-4. Anodizing or oxidizing:

Anodizing or oxidizing is another chemical method for treatment of dental implant surfaces, which is performed by electrochemical reaction. The aim of this treatment method is to increase the thickness of TiO2 layer in order to improve the surface characteristics of dental implants (29).

3-5. Plasma spraying:

Plasma spraying (PS) is one of the physical methods, which uses the atmospheric PS and vacuum plasma spraying to treat the implant surface. In this method the plasma torch is used to coat one of the three types of particles including titanium, calcium phosphate (CP), and hydroxyapatite (HA) (54). The titanium plasma spray (TPS) is effective in making surface roughness, which assists the rapid establishment of bone-implant-contact (BIC) (55). The rational for addition of hydroxyapatite or calcium and phosphor particles by plasma spraying method is similarity of these components with the basic structure of implant surrounding tissues (56). The plasma-sprayed hydroxyapatite (PSHA) was shown to better promote the osseointegration compared to uncoated surfaces (57).

The direct of effects of TPS and PSHA treatments on the angiogenesis events have not been evaluated. However, related to the titanium particles it should be noted that the titanium made healing caps affect the expression of VEGF, eNOS, iNOS, and nNOS in surrounding tissues (58). Several studies have also discussed the effects of calcium phosphate (CaP) ceramics such as hydroxyapatite on angiogenesis events (59-61). Chen *et al.* indicated that the calcium phosphate ceramics including hydroxyapatite can induce angiogenesis and neovascularization (59). Canuto *et al.* indicated that the nanocrystalline hydroxyapatite can enhance angiogenesis, osteogenesis, alveolar socket healing, and epithelialization through increased expression of BMP-4, BMP-7, VEGF, osteocalcin, and alkaline phosphatase (60). Similarly, Pezzatini *et al.* reported that the nanocrystals of HA are able to promote the expression of FGF-2, eNOS and promote the endothelial cells function during angiogenesis (61,62) ( Table 1 and  Table 1Continue).

3-6. Active drug incorporation:

The active drug incorporation is one of the recent treatment method for enhancement of dental implant surface characteristics. In this modification the bioactive elements such as bisphosphonate, simvastatin, and antibiotics are coated on the surface to promote the osseointegration process (29). The major aspect in adding bisphosphonate on the surface of dental implants is to prevent bone loss from the surrounding through time and increasing the density of alveolar bone (63). Or *et al.* showed that bisphosphonates such as Alendronate and Etidronate can induce inflammation by expression of cytokines, and inhibit angiogenesis events by decreasing the expression of FGF-2 and VEGF (64). Ribeiro *et al.* reported similar results using Alendronate and Zoledronate as these bisphosphonate drugs, exhibit anti-angiogenic effects by down-regulating VEGF expression. However, these authors presented that these bisphosphonates could up-regulate the expression of BMP-2, osteocalcin, osteoprotegerin, and alkaline phosphatase, which are effective in osteogenesis (65).

Simvastatin is another bioactive drug which is shown to be effective on bone formation and density (66,67). Several authors indicated that Simvastatin (3-hydroxy-3-methylglutaryl coenzyme A reductase inhibitor), which is cholesterol-lowering drug can promote angiogenesis events and endothelial cell functions (68-70). Gentamycin and Tetracycline-HCl are two types of antibiotic which have been coated on the surface of implants (29). Beside their antimicrobial activity, authors indicated that Tetracycline-HCl is able to enhance cellular attachment to the implant surface (71), and promote osseointegration through blood clot establishment on implant surface (72).

Lee *et al.* incorporated amoxicillin in nanotubes of TiO2 and concluded that this antibiotic can present a remarkable bactericidal effect in preventing the early failure of dental implants (73). He, Marneros indicated that doxycycline by affecting macrophages has anti-angiogenic impacts during neovascularization (74). Vancomycin is another antibiotic which Zhang *et al.* suggested for coating of titanium implant surface (75). Most of the antibiotic agents were indicated to have anti-angiogenic behavior (76-81). Meghari et al. showed that erythromycin has anti-angiogenic effect due to its action on endothelial cells proliferation (76). Other antibiotics such as ciprofloxacin (77,78), clindamycin (79), and minocycline (80,81) have also demonstrated anti-angiogenesis effects at applied sites.

3-7. Surface functionalizing:

The functionalization is a novel term in the dental implant surface modification era. In these surface treatments different growth factors and cytokines are utilized to enhance the biocompatibility and biological activity of implant surface (82). Several pro-angiogenic growth factors such as VEGF, EGF, insulin-like growth factors (IGF-1 and 2), platelet-derived growth factor (PDGF), TGF-β, and osteogenic proteins like BMP-2 and BMP-4 are suggested to be incorporated into the implant surface (29). Hossain *et al.* indicated that the hepatocyte growth factor (HGF) can be adsorbed to the hydroxyapatite surface of implants and promote osteogenesis and angiogenesis events during osseointegration process (83). Elkarargy reported that fibronectin can be used for surface functionalization in order to improve the osseointegration of dental implants (84).

Schliephake *et al.* showed that the coated recombinant human VEGF-165 on titanium surface could promote angiogenesis events either in immobilized or released form (85). Tan *et al.* reported that rhVEGF and antimicrobial peptide (AMP) coated on titanium stimulate the integration and decrease the infection risk (86). Poh *et al.* also suggested that the immobilized VEGF on titanium surface can promote the revascularization process around the implants which resulted in better integration (87) ( Table 1 and  Table 1Continue ,  Table 2).

As an eye to the future, it should be mentioned that several *in-vivo* and clinical studies are necessary to directly evaluate the effect of surface characteristics on angiogenesis process and to point out the specific effects and detailed mechanisms of the mentioned treatment methods on the angiogenesis process at the dental implant installation site.

## Conclusions

According to the point of views and aspects overviewed here the following conclusions can be drawn:

• The nano particles of TiO2 have anti-angiogenic behavior, while the ROS produced from the corrosion of titanium metal and its alloys act as a double edged sword, at low concentration is pro-angiogenic while at high concentration is anti-angiogenic by influencing endothelial cell function.

• The surface characteristics of dental implants have been well-discussed through the history and it was shown that surface topography and hydrophilicity are the most important and effective characteristics on angiogenesis and osteogenesis due to increasing the production of several pro-angiogenic growth factors. Meanwhile, there is still lack of knowledge on detail of mechanism of action of surface characteristic on angiogenesis.

• Among dental implant surface treatment methods, the sand-blasted large grit acid etched and acid-etched modifications were well-discussed methods. The current trends in surface treatments of dental implants are directed toward more complex and combination of methods and biomaterials in order to produce more bioactive and pro-angiogenic surfaces on dental implants.
